# Extraluminal recanalization for postoperative biliary obstruction using transseptal needle

**DOI:** 10.1186/s40792-020-01080-9

**Published:** 2020-12-03

**Authors:** Hiroki Horinouchi, Eisuke Ueshima, Keitaro Sofue, Shohei Komatsu, Takuya Okada, Masato Yamaguchi, Takumi Fukumoto, Koji Sugimoto, Takamichi Murakami

**Affiliations:** 1 Department of Radiology, Kobe University Graduate School of Medicine, 7-5-2, Kusunoki-cho, Chuo-ku, Kobe, Japan; 2 Department of Radiology, National Cerebral and Cardiovascular Center, Suita, Japan; 3 Department of Surgery, Division of Hepato-Biliary-Pancreatic Surgery, Kobe University Graduate School of Medicine, Kobe, Japan

**Keywords:** Biliary obstruction, Recanalization, Transseptal needle, Interventional radiology

## Abstract

**Background:**

Postoperative biliary strictures are commonly related to accidental bile duct injuries or occur at the site of biliary anastomosis. The first-line treatment for benign biliary strictures is endoscopic therapy, which is less invasive and repeatable. However, recanalization for biliary complete obstruction is technically challenging to treat. The present report describes a successful case of treatment by extraluminal recanalization for postoperative biliary obstruction using a transseptal needle.

**Case presentation:**

A 66-year-old woman had undergone caudal lobectomy for the treatment of hepatocellular carcinoma. The posterior segmental branch of the bile duct was injured and repaired intraoperatively. Three months after the surgery, the patient had developed biliary leakage from the right hepatic bile duct, resulting in complete biliary obstruction. Since intraluminal recanalization with conventional endoscopic and percutaneous approaches with a guidewire failed, extraluminal recanalization using a transseptal needle with an internal lumen via percutaneous approach was performed under fluoroscopic guidance. The left lateral inferior segmental duct was punctured, and an 8-F transseptal sheath was introduced into the ostium of right hepatic duct. A transseptal needle was advanced, and the right hepatic duct was punctured by targeting an inflated balloon that was placed at the end of the obstructed right hepatic bile duct. After confirming successful puncture using contrast agent injected through the internal lumen of the needle, a 0.014-in. guidewire was advanced into the right hepatic duct. Finally, an 8.5-F internal–external biliary drainage tube was successfully placed without complications. One month after the procedure, the drainage tube was replaced with a 10.2-F drainage tube to dilate the created tract. Subsequent endoscopic internalization was performed 5 months after the procedure. At the 1-year follow-up examination, there was no sign of biliary obstruction and recurrence of hepatocellular carcinoma.

**Conclusions:**

Recanalization using a transseptal needle can be an alternative technique for rigid biliary obstruction when conventional techniques fail.

## Background

Postoperative biliary strictures are commonly related to accidental bile duct injuries or occur at the site of biliary anastomosis during hepatobiliary surgery and liver transplantation [1]. The first-line treatment for benign biliary strictures is endoscopic therapy, which is less invasive and repeatable [1–4]. However, recanalization for biliary complete obstruction is technically challenging because sustained inflammation due to the biliary leakage and subsequent formation of fibrosis around the surgical site occur [2–4]. In the present case, we successfully treated a case of postoperative biliary obstruction via percutaneous approach by creating an extraluminal tract in the liver using a transseptal needle, which is a device to access the left atrium through the interatrial septum.

## Case presentation

**Fig. 1:**
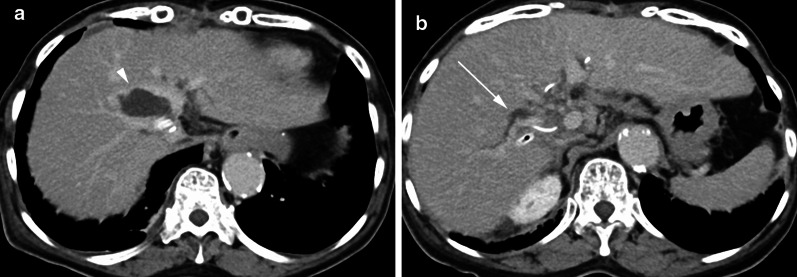
**a** Contrast-enhanced computed tomography after hepatectomy shows biliary leakage (arrowhead). **b** Intrahepatic bile duct of right lobe is dilated around the intraoperatively repaired site (arrow)

A 66-year-old woman had undergone caudal lobectomy for hepatocellular carcinoma. The posterior segmental branch of the bile duct was injured and repaired intraoperatively. A cystic duct tube (C-tube) was placed into the common bile duct, and a drainage tube was placed in the foramen of Winslow. Three months after the surgery, the patient presented a fever and increased levels of total bilirubin and alkaline phosphatase. Computed tomography (CT) showed biliary leakage and dilatation of the intrahepatic bile duct of right lobe around the intraoperatively repaired site (Fig. 1a, b).

**Fig. 2:**
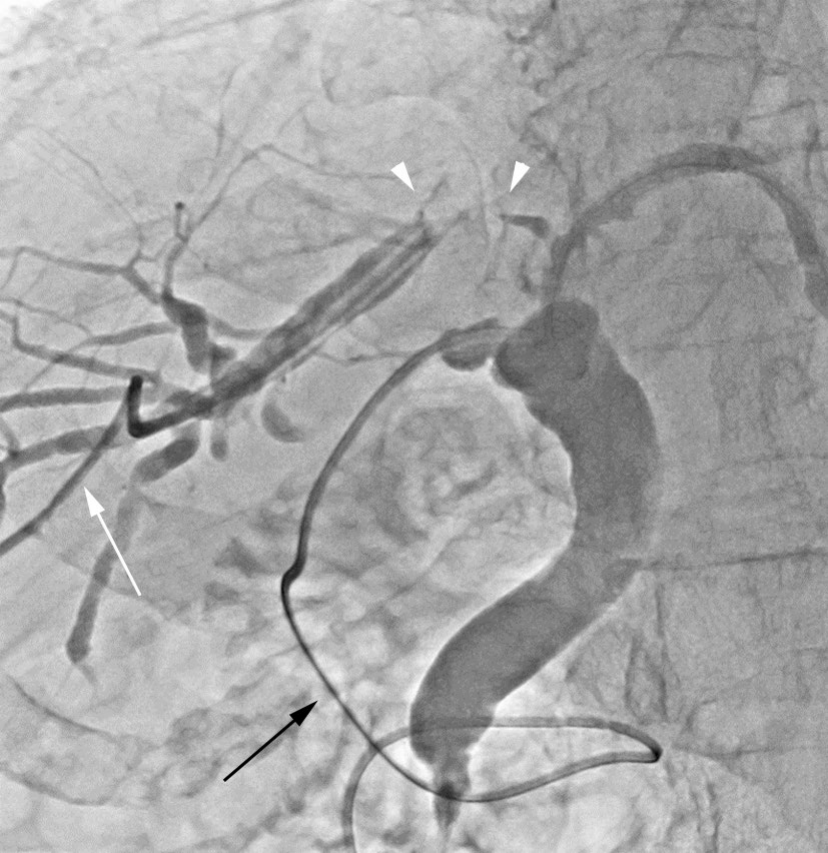
The cholangiogram via the PTBD catheter (white arrow) in the posterior segmental duct and the C-tube (black arrow) inside common bile duct demonstrates complete obstruction 15 mm in length in the right hepatic duct (white arrowheads)

A posterior segmental bile duct was percutaneously punctured under sonography, and a 7-F pigtail percutaneous transhepatic biliary drainage (PTBD) catheter (Hanako Medical, Saitama) was inserted. Simultaneous cholangiogram via the catheter and the C-tube showed complete obstruction 15 mm in length in the right hepatic duct (Fig. 2). Conventional endoscopic and percutaneous approaches with a guidewire failed to recanalize. The recanalization using the stiff edge of a 0.035-in. hydrophilic guidewire (Radifocus, Terumo, Tokyo) failed as well. Therefore, we proposed to use a transseptal needle to create the extraluminal tract after careful inspection that intrahepatic vasculature did not intervene between the extraluminal route on the contrast-enhanced CT images.

**Fig. 3:**
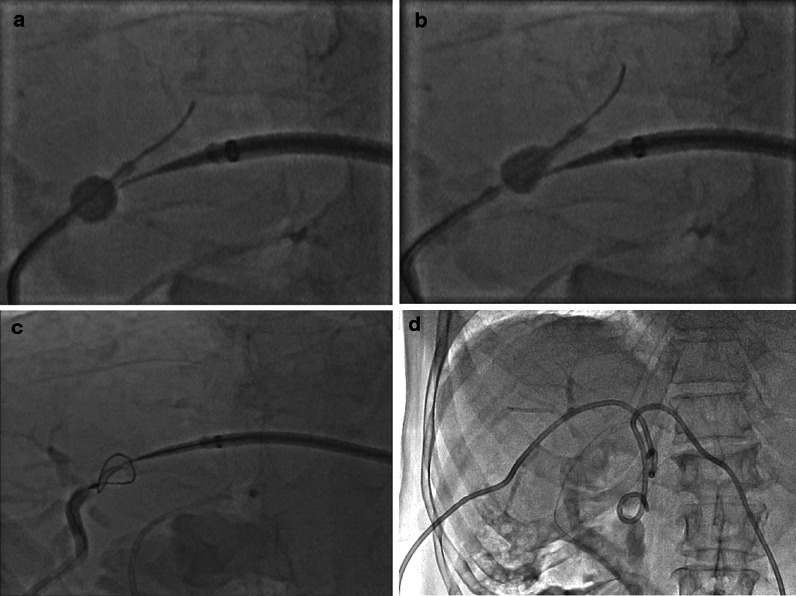
**a** A 6-F balloon catheter was placed at the distal end of right biliary obstruction, and a transseptal needle was inserted into the ostium of right hepatic duct through the left biliary access route. **b** The right hepatic duct was punctured by targeting the inflated balloon with the transseptal needle. **c** Following the successful puncture, a 0.014-in. guidewire was advanced through the transseptal needle lumen and pulled out by a loop snare catheter from the right biliary access route. **d** An internal–external PTBD catheter was placed into the common bile duct through the extraluminal recanalization tract

The procedure was performed under fluoroscopic guidance and local anesthesia. Firstly, a 6-F balloon catheter (Selecon MP Catheter II, Terumo, Tokyo) was placed at the end of the obstructed right hepatic bile duct through the initial PTBD route (Fig. 3a). Then, the left lateral inferior segmental duct was punctured using 21-gauge needle (PTCD needle; TOP Corporation, Tokyo) under cholangiogram guidance, and an 8-F transseptal sheath (SwartzTM; St. Jude Medical, Minnetonka, MN) was introduced into the ostium of right hepatic duct. A transseptal needle (BRK Transseptal Needle; St. Jude Medical, Minnetonka, MN) was advanced through the transseptal sheath, and the right hepatic duct was punctured by targeting the inflated balloon that was placed at the end of the obstructed right hepatic bile duct (Fig. 3b). After confirming successful puncture using contrast agent injected through the needle, a 0.014-in. guidewire (CHIKAI; ASAHI Intecc, Nagoya) was advanced into the right hepatic duct through the transseptal needle. The 0.014-in. guidewire was grasped with a loop snare catheter and pulled out through the right PTBD route, constructing a pull-through system (Fig. 3c). A 4.2-F Berenstein catheter (Hanako Medical, Saitama) was inserted along the pull-through guidewire from the right PTBD route through the created tract, and advanced to the common bile duct. After introducing a 0.035-in. stiff guidewire (Amplatz Extrastiff; COOK, Bloomington, IN), an 8.5-F drainage catheter (Dawson-Mueller Multipurpose Drainage Catheter, COOK, Bloomington, IN) with side holes was placed at the common bile duct through the tract (Fig. 3d). Finally, an internal–external PTBD catheter was successfully placed without complications.

One month after the procedure, the drainage tube was replaced with a 10.2-F drainage catheter (Dawson-Mueller Multipurpose Drainage Catheter) to maintain and dilate the created tract. Subsequent endoscopic internalization with a 7-F drainage catheter (Gadelius Medical K.K., Tokyo) was performed 5 months after the procedure. At the 1-year follow-up examination, there were no signs of biliary obstruction without recurrence of hepatocellular carcinoma.

## Discussion

Although revision surgery for postoperative biliary obstruction is a definitive treatment, it is technically challenging due to severe adhesion and has demonstrated a high morbidity and mortality rate. Percutaneous intervention is an alternative treatment option to avoid invasive surgery when endoscopic approach fails or is inaccessible due to choledochojejunostomy or hepaticojejunostomy reconstruction [1–4].

The extraluminal recanalization for biliary obstruction using the stiff edge of a guide wire has been reported in patients who had failed intraluminal recanalization with conventional techniques using a catheter and guide wire [3]. On the other hand, various techniques of recanalization with off-label use of devices such as radiofrequency puncture wires, yttrium aluminum garnet lasers or compressing magnets, have been reported [4–6]. In the present case, the transseptal needle was utilized to create the extraluminal tract for the following reasons: (1) the curved shape of the distal end of the device allows for easy adjustment of the puncture point; (2) the position of the tip can be confirmed by injecting contrast materials through the needle; and (3) a micro-guidewire can be subsequently advanced through the internal lumen [7]. Unusual employment of the transseptal needle has been reported in several situations to recanalize vascular occlusion or to create an extra-anatomical route [8–10]. As the use of transseptal needle carries the risk of injuring vessels which results in bleeding, the risk should be minimized by confirming vascular anatomy around the estimated puncture route on the preprocedural imaging.

## Conclusions

We reported a case of successful treatment for postoperative biliary obstruction by extraluminal recanalization using a transseptal needle. This technique can be an alternative intervention method for rigid biliary obstruction when conventional techniques fail.

## Data Availability

Not applicable.

## Abbreviations

C-tube: Cystic duct tube
CT: Computed tomography
PTBD: Percutaneous transhepatic biliary drainage

## References

[1] Gwon DI, Laash HU (2015). Radiological approach to benign biliary strictures. Gastrointest Interv.

[2] Rhee K, Jang SI, Lee D (2013). Recanalization of completely obstructed bilioenteric anastomoses using a needle knife puncture. Gastrointest Interv.

[3] Kim EH, Lee HG, Oh JS, Chun HJ, Choi BG (2018). Extraluminal Recanalization of Bile Duct Anastomosis Obstruction after Liver Transplantation. J Vasc Interv Radiol.

[4] Guimaraes M, Uflacker A, Schönholz C, Uflacker R (2010). Successful recanalization of bile duct occlusion with a radiofrequency puncture wire technique. J Vasc Interv Radiol.

[5] Endo M, Hashimoto M, Ohuchi Y, Ogawa T, Iwamoto A, Noguchi N (2017). Percutaneous Transhepatic Holmium:YAG Laser choledochojejunostomy for the recanalization of obstructed surgical anastomosis. J Vasc Interv Radiol.

[6] Oya H, Sato Y, Yamanouchi E, Yamamoto S, Hara Y, Kokai H (2012). Magnetic compression anastomosis for bile duct stenosis after donor left hepatectomy: a case report. Transplant Proc.

[7] O'Brien B, Zafar H, De Freitas S, Sharif F (2017). Transseptal puncture - Review of anatomy, techniques, complications and challenges. Int J Cardiol.

[8] Arabi M, Ahmed I, Mat'hami A, Ahmed D, Aslam N (2016). Sharp central venous recanalization in hemodialysis patients: a single-institution experience. Cardiovasc Intervent Radiol.

[9] Midulla M, Perini P, Sundareyan R, Lazguet Y, Dehaene A, Goyault G (2012). Transcatheter transcaval embolization of a type II endoleak after EVAR using a transseptal needle-sheath system. Vasc Endovascular Surg.

[10] Paolantonio G, Pietrobattista A, Parapatt GK, Liccardo D, Natali GL, Candusso M (2018). Successful percutaneous transhepatic recanalization of a completely obstructed hepatico-jejunal anastomosis in a child with liver transplantation: Unusual employment of the transseptal puncture system. Pediatr Transplant.

